# Wavelet multi-resolution approximation for multiobjective optimal control

**DOI:** 10.1371/journal.pone.0201514

**Published:** 2018-08-02

**Authors:** Wen Zou, Qingbin Zhang, Qingyu Gao, Zhiwei Feng

**Affiliations:** 1 College of Computer and Information Engineering, Hunan University of Commerce, Changsha, P.R.China; 2 College of Aerospace Science and Engineering, National University of Defense Technology, Changsha, P.R. China; Aristotle University of Thessaloniki, GREECE

## Abstract

A new sequential method based on multi-resolution approximation is proposed for solving computationally expensive multi-objective optimization problems. A traditional strategy is to decompose a multi-objective optimization problem into a number of single-objective optimization problems, whereby the PF can be regarded as a function of weights. Therefore, it is very natural to use wavelet multi-resolution approximation techniques for setting weight vectors. In our framework, the sequential approach starts with sampling aggressive functions on the initial coarsest grid with a few collocation points; once a rough PF is obtained, new points are automatically added on the basis of an adaptive wavelet collocation method. Therefore, the PF can be approximated with a relatively small number of weights. The efficiency of our method is demonstrated on two examples: a typical multi-objective optimization problem and an expensive multi-objective control optimal problem.

## Introduction

Multi-objective optimization is becoming increasingly important in many engineering fields. A multi-objective optimization problem (MOP) can have many, even an infinite number of Pareto optimal vectors. However, in some applications, the objective function evaluations could be extremely expensive computationally and/or financially [1]. For example, time-consuming simulation is often required in the optimal control of flexible spacecraft systems. It is practically important to develop algorithms which can approximate the Pareto front (PF) of a MOP within a tight budget on computational cost/time. Decomposition strategy is commonly used to solve multiobjective optimization. It constructs and solve a number of single objective optimization problems whose objective are weighted aggregations of the objective functions in the original MOP. The optimal solution of each problem is Pareto optimal to the MOP. All these optimal solutions form an approximation to the PF. However, a set of evenly distributed weight vectors may not results in a set of evenly distributed Pareto optimal solutions, and they may not approximate the PF very well [2, 3]. Therefore, a critical issue is how to choose a set of weight vectors. Kim proposed an adaptive weighted sum method for an MOP, in which the regions for further refinement are identified by assigning additional constraints [2, 3]. Due to its properties of localization in both space and scale, wavelet multi-resolution approximation has been recently adopted to obtain compact representations of integral and differential operators in many physical problems, especially in solving partial differential equations (PDEs) [4–8]. Since the goal of multi-objective optimization is to find the well-represented Pareto optimal solutions, it is very natural to use such approximation techniques for approximate the entire PF. To the best of our knowledge, our work in this paper is the first attempt to adopt a wavelet multi-resolution method for approximating the PF of expensive multi-objective optimal control problems. The basic idea of our proposed method is to embed multi-resolution approximation techniques into the MOP decomposition strategy using an adaptive wavelet collocation method [9,10].

The remainder of this paper is organized as follows. Section II briefly introduces second-generation wavelets and demonstrates their ability on a typical example. Section III presents the traditional weighted approach for solving an MOP and describes the proposed framework for approximating the PF of an expensive multi-objective optimal control problem (MOCP) on the basis of an adaptive wavelet collocation method. Section IV demonstrates the efficiency of our scheme using two typical examples. Finally, Section 5 concludes the paper.

## Approximation of second-generation wavelets

Wavelet decomposition is to represent a function using wavelet basis functions that are localized in both space and level. In the second-generation multi-resolution analysis, a function *f* ∈ *L*^2^ can be approximated on a certain level of *J* as follows:

$$f^{J}(t)=\sum_{k\in K^{0}}c_{k}^{0}\phi_{k}^{0}(t)+\sum_{j=0}^{J-1}\sum_{l\in K^{j}}d_{l}^{j}\psi_{l}^{j}(t)$$ (1)

where $\phi_{k}^{0}(t)$ is the scaling function on the coarsest level of resolution, $\psi_{l}^{j}(t)$ and $d_{l}^{j}$ denote the wavelet coefficients, the superscript *j* is the level of resolution, the subscript *l* is related to the location in the physical space at each level, $\psi_{l}^{j}(t)$ is a wavelet function, *J* determines the finer scale, *K*^*j*^ is the index set.

The wavelet $\psi_{l}^{j^{\prime }}(t)$ belongs to the adjacent zone of wavelet $\psi_{l}^{j}(t)$ if the following relations are satisfied [4–8]:

$$\begin{array}{l} \left|j-j^{\prime }\right|<L\\ \left|2^{j-j^{\prime }}-k\right|<M \end{array}$$ (2)

where *L* determines the extent to which coarser and finer scales are included in the adjacent zone, and *M* defines the width of the adjacent zone on the same level.

Given a threshold value *ε*, the approximation can be rewritten as a sum of two terms composed of wavelets whose coefficients are above and below the given threshold *ε* [8]:

$$f^{J}=f_{>}^{J}+f_{\leq }^{J}$$ (3)

where

$$f_{>}=\sum_{k\in K^{0}}c_{k}^{0}\varphi_{k}^{0}(t)+\sum_{j=0}^{J-1}\sum_{\begin{array}{l} h\in L^{j}\\ \left|d_{h}^{j}\right|>\varepsilon \end{array}}^{}d_{h}^{j}\psi_{h}^{j}(t)$$ (4)

$$f_{\leq }=\sum_{j=0}^{J-1}\sum_{\begin{array}{l} h\in L^{j}\\ \left|d_{h}^{j}\right|\leq \varepsilon \end{array}}^{}d_{h}^{j}\psi_{h}^{j}(t)$$ (5)

To illustrate the approximation ability of the wavelet multi-resolution decomposition, the following function is used as an example

$$f(t)=\left[0.3t^{2}\text{cos}(24\pi t+\frac{12\pi }{5})+0.6t\right]\text{cos}(6\pi t+\frac{3\pi }{5})\;\left(0\leq \text{t}\leq 1\right).$$ (6)

**Fig 1:**
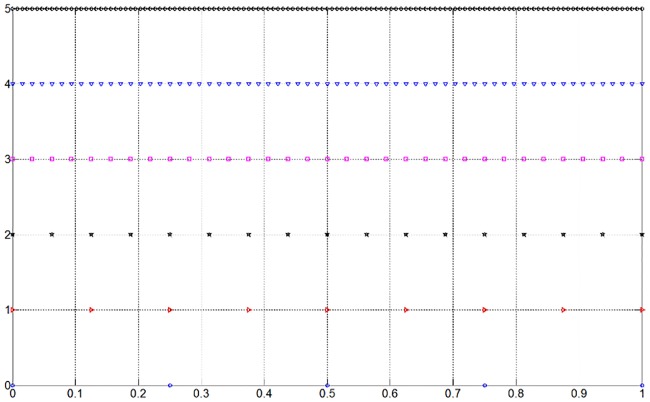
Dyadic grids on [0, 1].

**Fig 2:**
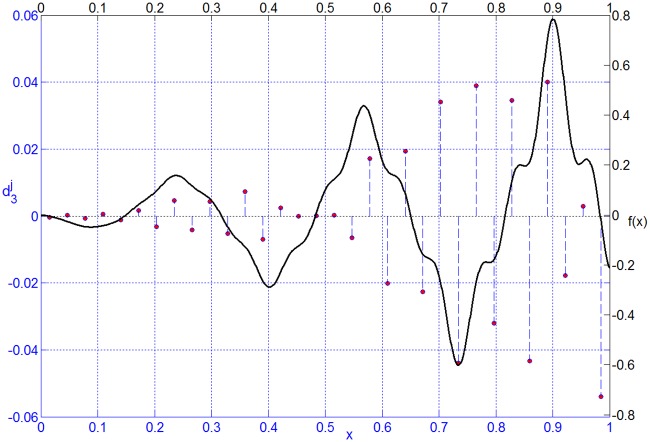
Distribution of wavelet coefficients.

The distribution of collocation points on the interval [0, 1] for level *j* = 0,1,⋯,5 is shown in Fig 1. The function and its wavelet coefficients for the third level are shown in Fig 2. It is obvious that the wavelet coefficients are small in the smooth region and large in the region with sharp features. Noting that setting a wavelet coefficient to be zero is equivalent to discarding the corresponding collocation point. A function can be approximated by deleting wavelets whose coefficients are lower than the given threshold.

## General framework

### 3.1. Multiobjective optimization problem and decomposition

A multi-objective optimization problem (MOP) can be stated as follows [11–12]:

$$\begin{array}{l} \text{min}\;F\left(x\right)={\left(f_{1}\left(x\right),\ldots f_{m}\left(x\right)\right)}^{T}\\ subject\;to\;x\in \Omega \end{array}$$ (7)

where Ω is the decision (variable) space, *F*:Ω→*R*^*m*^ consists of *m* real-valued objective continuous functions, and *R*^*m*^ is the objective space. A point x* ∈ Ω is said to be Pareto optimal if there is no *x* ∈ Ω such that *F*(*x*) dominates *F*(x*). The set of all the Pareto optimal points is called the Pareto set (PS). The set of all the Pareto optimal objective vectors, F = {*F*(*x*) ∈ *R*^*m*^|*x* ∈ *PS*}, is called the Pareto front (PF).

Decomposition is a basic strategy in multiobjective optimization. For the sake of simplicity, the weighted sum approach is employed in this paper. Let **λ** = [*λ*_1_,⋯,*λ*_*m*_] be a weight vector, i.e., *λ*_*i*_ ≥ 0 for all *i* = 1,⋯,*m* and $\sum_{i=1}^{m}\lambda_{i}=1$. Then, the optimal solution to the scalar optimization problem [11,12]

$$\begin{array}{l} minimize\;g^{ws}\left(x|\lambda \right)=\sum_{i=1}^{m}\lambda_{i}f_{i}\left(x\right)\\ subject\;to\;x\in \Omega \end{array}$$ (8)

is a Pareto optimal point of Eq (7). For each optimal point, there exists a weight vector *λ* such that x* is the optimal solution of Eq (8), and each optimal solution of Eq (8) is a Pareto optimal point if the PF is convex. One can obtain a set of different Pareto optimal solutions by solving a set of single-objective optimization problems with different weights.

### 3.2. Framework for solving multi-objective optimal control problem (MOCP)

A multi-objective optimal control problem (MOCP) can be defined as follows [13]. Determine the state **x**(*t*), control **u**(*t*), initial time *t*_0_, and terminal time *t*_*f*_ for minimizing the following Mayer performance indices:

$$J_{i}=\Phi_{i}\left[x(t_{0}),t_{0},x(t_{f}),t_{f}\right]+\int_{t_{0}}^{t_{f}}L_{i}\left[x(t),u(t),t\right]dt\;\left(i=1,\cdots ,m\right)$$ (9)

subject to the dynamic constraints

$$\dot{x}(t)=f\left[x(t),u(t),t\right],$$ (10)

the path constraints

$$C_{\text{min}}\leq C\left[x(t),u(t),t\right]\leq C_{\text{max}}$$ (11)

and bound conditions

$$\Psi_{\text{min}}\leq \Psi \left[x(t_{0}),t_{0},x(t_{f}),t_{f}\right]\leq \Psi_{\text{max}}$$ (12)

The above problem is often very difficult in engineering areas, since each solution evaluation can be very computationally expensive. In this paper, we use the decomposition strategy for handling it. More specifically, we consider:

$$\text{minimize}\;g^{ws}\left(x|\lambda \right)=\sum_{i=1}^{m}\lambda_{i}J_{i}\left(x,u,t_{0},t_{f}\right)$$ (13)

subject to the constraints given by Eqs (10)–(12).

For a given weight, we can use a standard single objective optimizer for solving the above problem and produce one Pareto optimal solution. The PF of the MOCP can be regarded as a function of the weight. We use the wavelet multi-resolution approximation for choosing weight vectors. Our basic idea is to first obtain an initial approximation of the PF using a coarsest grid with only a few weights, and to add new weight vectors successively in the regions where the PF has sharp variations. For the sake of notational simplicity, our proposed method is described in the following for the bi-objective optimal control problem. We should point out that it can be extended to more than two objectives by simply employing multi-dimensional wavelets. Our method consists of the following five steps.

**Step 1** Initialization
  - the threshold *ε*
  - adjacent wavelet parameters *L* and *M*
  - resolution level *j* = 0,1,⋯,*J*_max_
  - *G*_*iter*_ = *G*_*add*_ = *G*^1^
**Step 2** Solve the scalar optimal sub-problem given by Eq (13) for all *λ*^*j*^ ∈ *G*_*add*_ and define the set $S_{i}=\left\{J_{i}^{j}|\lambda^{j}\in G_{iter}\right\}\;for\;i=1,\cdots ,m$
**Step 3** Select the new weights that would be involved in the next step.
  Set *G*_*adj*_ = *ϕ*; then, for all *i* = 1,⋯,*m*
**Step 3.1** Compute the wavelet coefficients using the forward wavelet transform for the sample data *S*_*i*_ with respect to the weights, and analyze the wavelet coefficient $d_{k}^{j}$.
**Step 3.2** Select the unsmooth region where the magnitude of the wavelet coefficients is larger than the threshold *ε*.
**Step 3.3** According to the parameters *L* and *M*, collect all the adjacent wavelets related to the above-mentioned unsmooth region, denote these collocation points as $G_{i}^{adj}$, and define $G_{add}=G_{add}\cup G_{i}^{adj}$.
**Step 4** Increase *j* by 1; if *j* < *J*_max_, set *G*_*iter*_ = *G*_*iter*_ ∪ *G*_*add*_ and go to Step 2; otherwise, terminate the algorithm and move on to the next step.
**Step 5** According to the final sample value of *S*_*i*_ on the final grid points *G*_*iter*_, approximate the PF on $G^{J_{\text{max}}}$ for the highest level.

**Fig 3:**
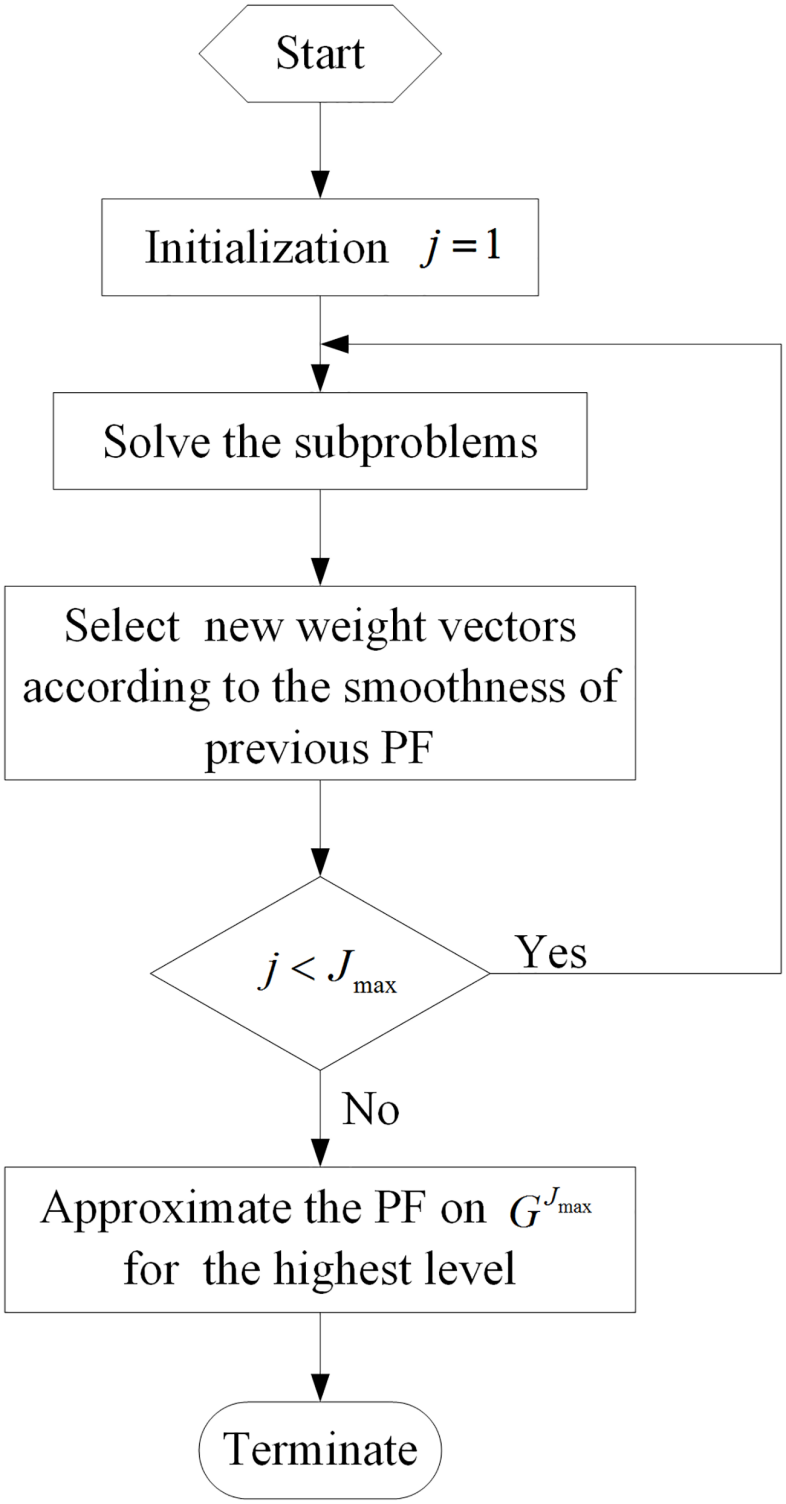
Flowchart of the sequential scheme.

As shown in Fig 3, this sequential process can be simply illustrated by a flowchart.

Some remarks on this scheme are given below.

- In Step 1, for *j* = 0,1,⋯,*J*_max_, a set of dyadic grids on the interval [0, 1] can be constructed. It is clear that each point $t_{k}^{j}\in G^{j}$ is equivalent to a unique weight for the decomposition strategy.
- In Step 2, the MOCP is solved just for the weights from *G*_*add*_.
- In Step 3, according to the values *S*_*i*_ at *G*_*iter*_ for each objective, the unsmooth region is identified by the given threshold *ε*; then, the adjacent wavelet collocations are selected according to *L* and *M*. It should be noted that the parameters *ε*, *L*, and *M* should be selected on the basis of some experience, because this sequential scheme is a type of heuristic algorithm to some extent.
- In Step 5, the entire PF can be approximated according to the final set $S_{J_{\text{max}}}$ on *G*_*iter*_ with a relatively small number of weights.

With such an algorithm the grid of collocation points adapts dynamically to follow local structures that appear in solution of PF. Hence, it should be noted that overall computational cost of the algorithm is related with the size of subproblems (or the size of population) and solving strategy of optimal control problem.

## Simulation

Two typical examples are used to investigate the performance of the proposed approach. All the single-objective optimization problems are solved using sequential quadratic programming in MATLAB^®^ [14].

### 4.1. Multi-objective optimization problem

The multi-objective optimization problem investigated can be stated as follows [2,3]:

$$minimize\;\{\begin{array}{c} f_{1}=x_{1}^{2}+x_{2}^{2}+x_{3}^{2}+x_{4}^{2}+x_{5}^{2}\\ f_{2}=3x_{1}^{}+2x_{2}^{}-\frac{x_{3}}{3}+{\left(x_{4}^{}-x_{5}^{}\right)}^{2} \end{array}$$ (14)

$$subject\;to\;\left\{ \begin{array}{l} x_{1}^{}+2x_{2}^{}-x_{3}^{}+0.5x_{4}^{}+x_{5}^{}=2\\ 4x_{1}^{}+2x_{2}^{}-0.8x_{3}^{}+0.6x_{4}^{}+0.5\\ x_{1}^{2}+x_{2}^{2}+x_{3}^{2}+x_{4}^{2}+x_{5}^{2}\leq 10 \end{array}\right.$$ (15)

**Fig 4:**
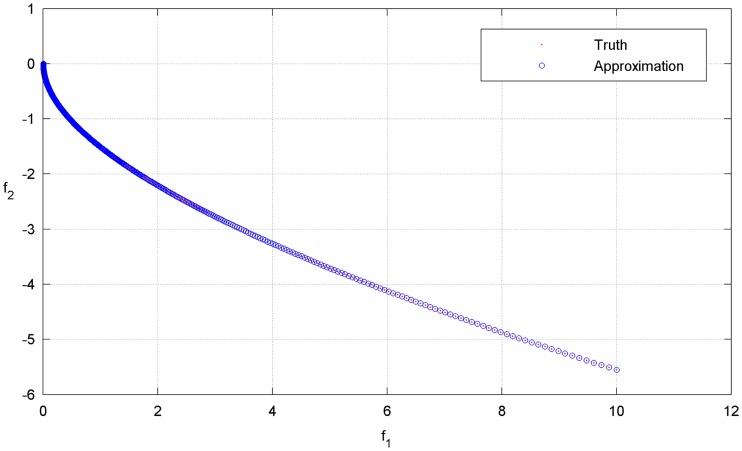
Approximation and ground truth of the PF.

**Fig 5:**
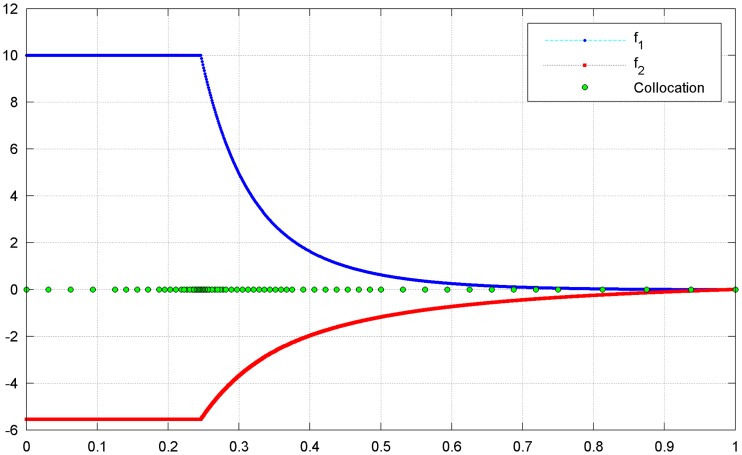
Optimal objectives and the collocation points.

**Fig 6:**
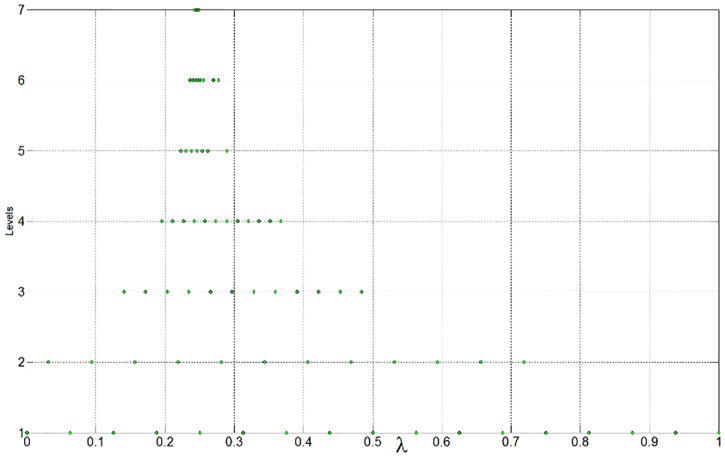
Distribution of the collocation points on each level.

**Fig 7:**
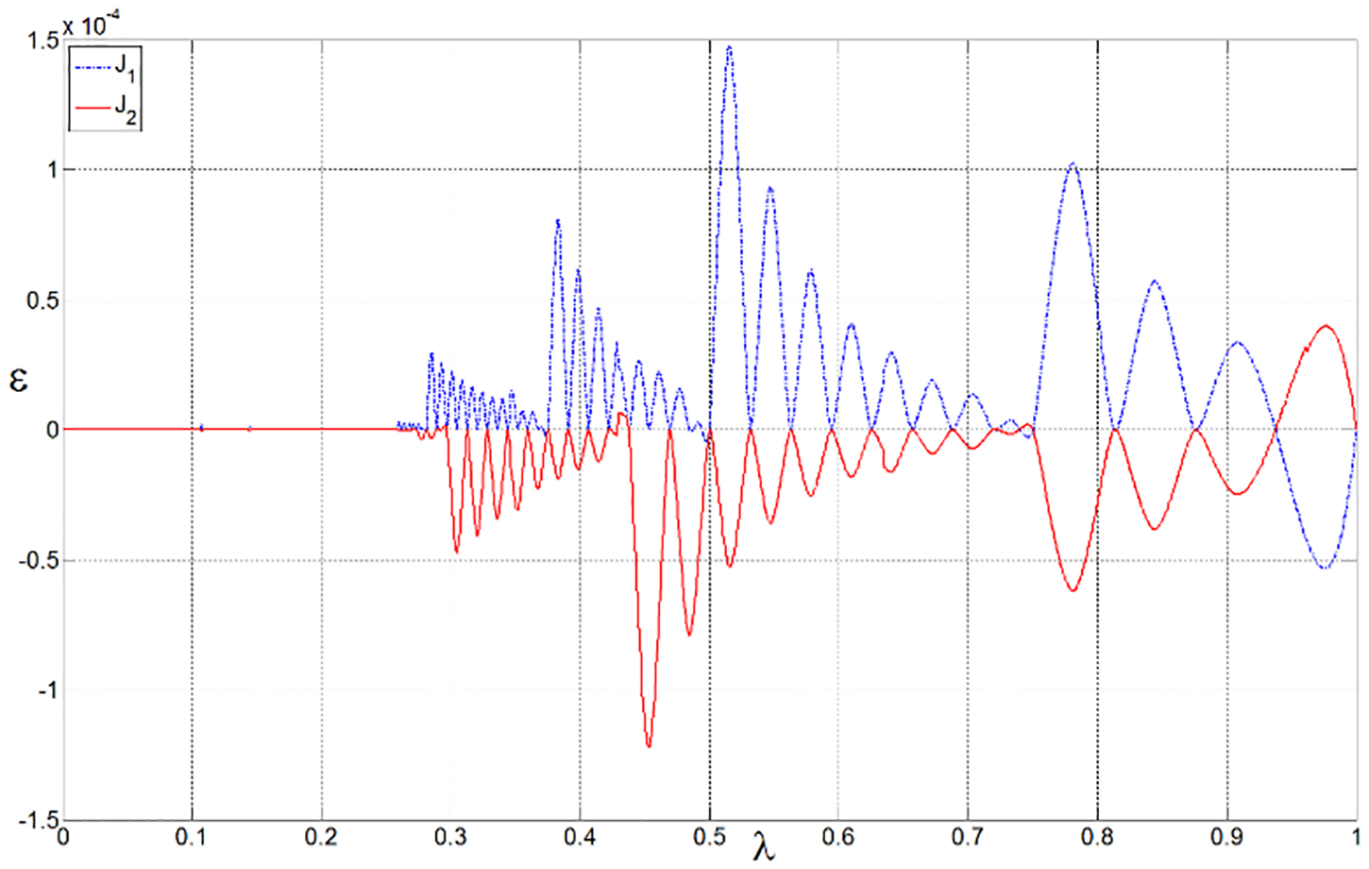
Approximation error.

The PF of this problems is convex, but its curvature is not uniform [2,3]. In the proposed multi-resolution approximation approach, a set of dyadic grids is first constructed on the interval [0, 1], with 17 points on the coarsest level and 1025 points on the highest level. Note that all these wavelet collocation points are equivalent to the weights used in the sub-problems of Eq (13). For comparison, the PF obtained from the 1025 uniform weights on the highest level is regarded as the true PF. The parameters in the optimal operation are set as *ε* = 0.0002 and *L* = *M* = 1. As shown in Fig 4 (see S1 Fig), the approximation is in excellent agreement with the ground truth. As stated in Section 3.1, the conventional strategy is to decompose an MOP into a limited number of scalar optimization sub-problems, and the number of these sub-problems is exactly equal to the number of weights. Therefore, the computational resources are mainly determined by the number of weight vectors. A small number of weights involved in the decomposition approach means that less computational resources would be required in the optimization process. In this example, only 71 collocation points (weights) are required to approximate the PF; thus, resource usage is reduced by nearly 93%. The optimal objectives as well as the involved collocation points are shown in Fig 5(see S1 Fig). The distribution of the collocation points on each level is shown in Fig 6 (see S1 Fig). It can be seen that most of the weights are allocated to the regions where the components of the PF have sharp variations, while only a few weights are allocated to the smooth regions. The approximation error of the optimal objectives is shown in Fig 7 (see S1 Fig).

### 4.2. MOCP in flexible spacecraft system

**Fig 8:**
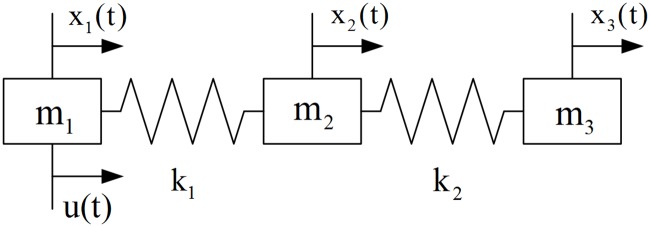
Three-mass-spring system.

As shown in Fig 8, a flexible spacecraft with a rigid mode and two flexible modes [15] is applied to further illustrate the efficiency of our proposed method. The nominal parameter values are assumed as *m*_1_ = *m*_2_ = *m*_3_ = 1 and *k*_1_ = *k*_2_ = *k*_3_ = 1 with appropriate units, and the time is measured in seconds. The control force *u*, which is bounded as |*u*|≤1, is applied to body 1. Finally, the time/fuel optimal control problem can be formulated as follows:

$$\text{min}\{\begin{array}{c} J_{1}=\int_{0}^{t_{f}}dt=t_{f}\\ J_{2}=\int_{0}^{t_{f}}\left|u\right|dt \end{array}$$ (16)

subject to the dynamics equation

$$M=\left[\begin{array}{ccc} m_{1} & & \\ & m_{2} & \\ & & m_{3} \end{array}\right]\left[\begin{array}{c} {\ddot{x}}_{1}\\ {\ddot{x}}_{2}\\ {\ddot{x}}_{3} \end{array}\right]+\left[\begin{array}{ccc} k_{1} & -k_{1} & \\ -k_{1} & k_{1}+k_{2} & -k_{2}\\ & -k_{2} & k_{2} \end{array}\right]\left[\begin{array}{c} x_{1}\\ x_{2}\\ x_{3} \end{array}\right]=\left[\begin{array}{c} 1\\ 0\\ 0 \end{array}\right]u$$ (17)

where *x*_1_, *x*_2_, and *x*_3_ are the positions of bodies 1, 2, and 3, respectively. In addition, the boundary conditions for the rest-to-rest maneuver are given by

$$x_{1}(0)=x_{2}(0)=x_{3}(0)=0\;x_{1}(t_{f})=x_{2}(t_{f})=x_{3}(t_{f})=1$$ (18)

$${\dot{x}}_{1}(0)={\dot{x}}_{2}(0)={\dot{x}}_{3}(0)=0\;{\dot{x}}_{1}(t_{f})={\dot{x}}_{2}(t_{f})={\dot{x}}_{3}(t_{f})=0$$ (19)

Based on the weighted sum decomposition approach, a single-objective optimal control problem can be constructed as follows:

$$minimize\;J(\lambda )=\int_{0}^{t_{f}}(\lambda +(1-\lambda )\left|u\right|)dt\;\lambda \in [0,1]$$ (20)

subject to the constraints given by Eqs (17)–(19).

**Fig 9:**
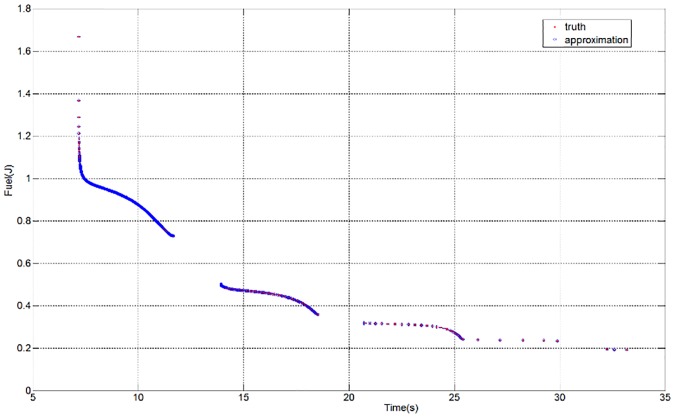
PF of time/fuel optimal control problem.

**Fig 10:**
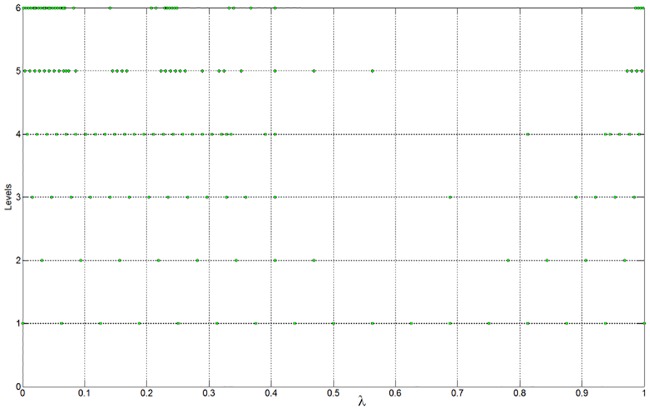
Distribution of the involved collocation points on each level.

**Fig 11:**
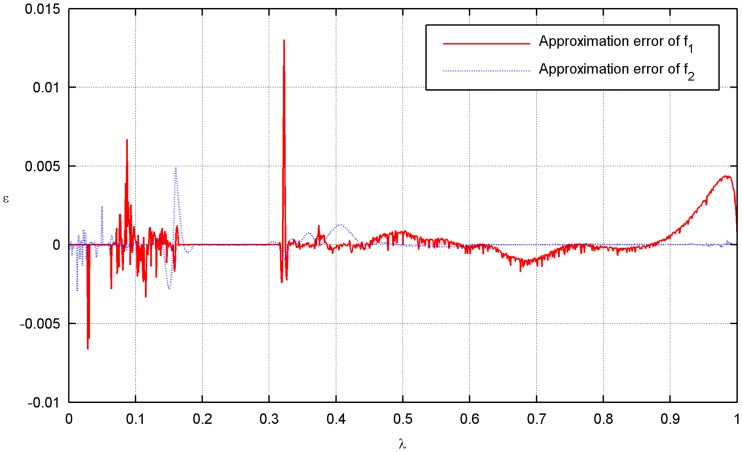
Approximation error of the optimal objectives.

In the optimal process, the set of dyadic grids and the true PF are constructed as discussed in the previous example. The parameters in the optimal operation are set as *ε* = 0.005 and *L* = *M* = 1. A comparison between the approximation and the ground truth is shown in Fig 9 (see S2 Fig). There exist some discontinuities in the PF, which are caused by the physical constraints of the flexible mode. The distribution of the collocation points on each level is shown in Fig 10 (see S2 Fig). As expected, the approximation can be obtained with only 173 weights; thus, resource usage is reduced by nearly 83%. The approximation error is shown in Fig 11(see S2 Fig).

## Conclusion

Using the traditional decomposition approach, this paper proposed a multi-resolution method for solving expensive MOCP arising from practical engineering applications. In our proposed method, an adaptive multi-resolution approximation strategy is embedded into the decomposition approach. It starts with a few weights according to the initial coarsest grid; then, the approximation can be refined successively by locally increasing the accuracy in the unsmooth vicinity associated with large wavelet coefficients. Our method can automatically generate non-uniform weights to approximate the entire PF with minimal computational effort. Therefore, one can obtain the PF accurately and efficiently with a relatively small number of weights. The presented examples demonstrated that the proposed method can efficiently obtain the PF using the traditional decomposition strategy. Currently work is under way to develop an adaptive algorithm in the design of multi-objective optimal homing trajectory of parafoil system.

## Supporting information

S1 Fig Result of simulation example 1. (XLSX)

S2 Fig Result of simulation example 2. (XLSX)

## References

[1] Zhang Q, Liu W, Tsang E, Virginas B. Expensive Multiobjective Optimization by MOEA/D With Gaussian Process Model. IEEE Transactions on Evolutionary Computation. 2010;14(3):456–74.

[2] Kim IY, Weck OLD. Adaptive weighted-sum method for bi-objective optimization: Pareto front generation. Structural & Multidisciplinary Optimization. 2013;29(2):149–58.

[3] Kim IY, Weck OLD. Adaptive weighted sum method for multiobjective optimization: a new method for Pareto front generation. Structural & Multidisciplinary Optimization. 2006;31(2):105–16.

[4] Kai S, Vasilyev OV. Wavelet Methods in Computational Fluid Dynamics. Annual Review of Fluid Mechanics. 2009;42(42):473–503.

[5] Grossmann A, Morlet J. Decomposition of Hardy function into square integrable wavelets of constant shape. SIAM J Math Anal; 1984.

[6] Vasilyev OV, Paolucci S, Sen M. A Multilevel Wavelet Collocation Method for Solving Partial Differential Equations in a Finite Domain. Journal of Computational Physics. 1995;120(1):33–47.

[7] Alam JM, Kevlahan KR, Vasilyev OV. Simultaneous space–time adaptive wavelet solution of nonlinear parabolic differential equations. Journal of Computational Physics. 2006;214(2):829–57.

[8] Vasilyev OV, Bowman C. Second-Generation Wavelet Collocation Method for the Solution of Partial Differential Equations. Journal of Computational Physics. 2000;165(2):660–93.

[9] Zhang Q, Feng Z, Tang Q, Zhang Y. An adaptive wavelet collocation method for solving optimal control problem. Proceedings of the Institution of Mechanical Engineers Part G Journal of Aerospace Engineering. 2014.

[10] Zhang Q, Feng Z, Tang Q, Malcolm M. A spline wavelet collocation method for the optimal control of flexible spacecraft. Proceedings of the Institution of Mechanical Engineers Part G Journal of Aerospace Engineering. 2015;229(1):163–71.

[11] Vasilyev OV, Paolucci S, Sen M. A Multilevel Wavelet Collocation Method for Solving Partial Differential Equations in a Finite Domain. Journal of Computational Physics. 1995;120(1):33–47.

[12] Li H, Zhang Q. Multiobjective Optimization Problems With Complicated Pareto Sets, MOEA/D and NSGA-II. IEEE Transactions on Evolutionary Computation. 2009;13(2):284–302.

[13] Rao AV. A Survey of Numerical Methods for Optimal Control. Advances in the Astronautical Sciences. 2010;135(1).

[14] Gill PE, Murray W, Saunders MA. SNOPT: An SQP Algorithm for Large-Scale Constrained Optimization: Society for Industrial and Applied Mathematics; 2002 99–131.

[15] Wie B. Space Vehicle Dynamics and Control, Second Edition. Embo Journal. 1971;32(12):1745–60.

